# The evolution, facilitators, barriers, and additional activities of acute flaccid paralysis surveillance platform in polio eradication programme Bangladesh: a mixed-method study

**DOI:** 10.1080/16549716.2024.2370096

**Published:** 2024-06-27

**Authors:** Humayra Binte Anwar, Yameen Mazumder, Sanjana Nujhat, Bushra Zarin Islam, Anna Kalbarczyk, Olakunle Alonge, Malabika Sarker

**Affiliations:** a BRAC James P Grant School of Public Health, BRAC University, Dhaka, Bangladesh; b Johns Hopkins Bloomberg School of Public Health, 615 N. Wolfe Street, Baltimore, MD, USA; c Sparkman Center for Global Health, School of Public Health, University of Alabama at Birmingham (UAB), Birmingham, AL, USA; d Department of Behavioral and Social Sciences, Brown University School of Public Health, Providence, RI, USA

**Keywords:** Acute Flaccid Paralysis (AFP) surveillance, polio eradication, vaccine-preventable disease, partnership, Bangladesh

## Abstract

**Background:**

The Global Polio Eradication Initiative (GPEI) helped develop the standard acute flaccid paralysis surveillance (AFP) system worldwide, including, knowledge, expertise, technical assistance, and trained personnel. AFP surveillance can complement any disease surveillance system.

**Objective:**

This study outlines AFP surveillance evolution in Bangladesh, its success and challenging factors, and its potential to facilitate other health goals.

**Methods:**

This mixed-method study includes a grey literature review, survey, and key informant interviews (KIIs). We collected grey literature from online websites and paper documentation from GPEI stakeholders. Online and in-person surveys were conducted in six divisions of Bangladesh, including Dhaka, Rajshahi, Rangpur, Chittagong, Sylhet, and Khulna, to map tacit knowledge ideas, approaches, and experiences. We also conducted KIIs, and Data were then combined on focused emerging themes, including the history, challenges, and successes of AFP surveillance programme.

**Results:**

According to the grey literature review, survey, and KII, AFP surveillance successfully contributed to decreasing polio in Bangladesh. The major facilitating factors were multi-sectoral collaboration, Surveillance Immunization Medical Officer (SIMO) network activities, social environment, community-based surveillance, and promising political commitment. On the other hand, high population growth, hard-to-reach areas, people residing in risky zones, and polio transition planning were significant challenges. Bangladesh is also utilizing these polio surveillance assets for other vaccine-preventable diseases.

**Conclusion:**

As the world is so close to eradicating polio, the knowledge, and other assets of the AFP surveillance, could be used for other health programmes. In addition, its strengths can be leveraged for combating new and emerging diseases.

## Paper Context

- **Main findings:** The research found that Bangladesh has achieved a world-standard surveillance system, with facilitating factors including multi-sectoral collaboration, GPEI partners, and political and community support. However, high population growth, hard-to-reach areas and people, and polio transition planning were found to be challenges.
- **Added knowledge:** In addition, Bangladesh is now utilizing these polio surveillance assets to monitor other vaccine-preventable diseases.
- **Global health impact for policy and action:** Since polio is still a threat to some LMICs, the knowledge gained from AFP surveillance of Bangladesh could assist those countries in eradicating the cases of polio from the earth and serve VPDs and other health programmes as well.

## Background

At the 41^st^ World Health Assembly (WHA) in 1988, Global Polio Eradication Initiative (GPEI) was launched to eradicate poliomyelitis, a potentially fatal infectious disease caused by three viral serotypes by the year 2000 [1]. The World Health Organization (WHO), in collaboration with Rotary International, the US Centers for Disease Control and Prevention (CDC), the United Nations Children’s Fund (UNICEF), and Global Alliance for Vaccine Initiative jointly led the goal of polio eradication [2]. One of the critical strategies for GPEI was establishing a unique surveillance system to detect all Acute Flaccid Paralysis (AFP) cases in children in every country [3]. AFP is the sudden onset of weakness or paralysis of limbs often associated with fever in children under fifteen years of age that occurs in polio and other diseases with a similar features, such as Guillain-Barre syndrome, transverse myelitis, traumatic neuritis meningitis, encephalitis, and brain tumours [4,5]. AFP surveillance has both passive (reports from field) and active surveillance (active search by surveillance teams) systems. AFP surveillance quality depends on early detection and reporting, specimen collection and reverse cold chain, prompt transportation of specimens to the laboratory, and timely reporting of results [5,6]. Despite decline in worldwide cases, Afghanistan, and Pakistan – have yet to eradicate wild polio. According to WHO, circulating vaccine-derived poliovirus type-2 (cVDPV2) cases are spreading globally rapidly and the number of cVDPV2 in 2020 was 1,009, 254% higher than in 2019 [7].

The knowledge assets including, evolution, and innovations learned from the AFP surveillance system can complement other surveillance activities to detect disease outbreaks in the community [8]. Many countries now use the AFP surveillance system, to monitor other vaccine-preventable diseases (VPD) [9,10]. Similarly, in some African countries of AFP surveillance teams have been deployed to identify active case searches for COVID-19 [11].

Bangladesh has adopted a polio eradication goal since 1995. It has implemented four basic strategies following WHO’s suggestion, including i) Routine immunization (RI), ii) Supplementary immunization activities (SIAs), iii) AFP surveillance, and iv) Mop-up campaigns [12,13]. The country with WHO South-East Asia Region achieved polio-free status on 27 March 2014 [14], and since maintaining all ten key performance indicators for AFP surveillance [12]. Bangladesh has also integrated other VDP surveillances into the AFP surveillance [15]. An evaluation of the AFP surveillance system showed that the system correctly identified children with polio and non-polio diseases and guided mop-up campaigns to raise awareness and promote polio vaccination [12]. The AFP surveillance team in Bangladesh has also been serving as frontline health workers for the COVID-19 response [16]. Thus, there is a necessity to synthesize the vast array of AFP surveillance activities; however, no study has taken a comprehensive and systematic approach to capturing the rich knowledge surrounding the AFP surveillance system in Bangladesh. This paper describes the evolution of AFP surveillance in Bangladesh, its facilitators, and barriers and additional activities leveraged from implementing the programme.

## Methods

**Figure 1.**
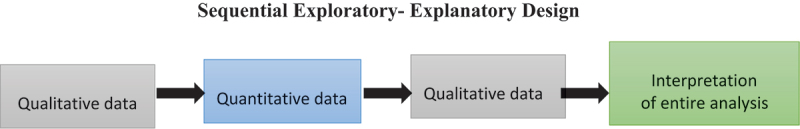
Sequential exploratory-explanatory mixed-method for the current study.

This study is a part of the ‘Synthesis and Translation of Research and Innovations from Polio Eradication’ (STRIPE) project, which is a consortium of seven international academic partners, including the James P Grant School of Public Health, BRAC University, Dhaka, Bangladesh, and is led by the Johns Hopkins Bloomberg School of Public Health (JHSPH) [17]. A sequential explanatory mixed-method study design (Figure 1) was used to map, synthesize, and disseminate lessons learned from the global polio eradication effort in Bangladesh [18].

We conducted a grey literature review regarding polio eradication programme in Bangladesh, which was used to develop the survey questionnaire, followed by key informant interviews (KII) guidelines to complement the mixed-method findings.

For grey literature, a team of two independent researchers explored Government of Bangladesh websites with permission and related organizations such as Rotary International, UNICEF, BMGF, WHO, GAVI, and the International Centre for Diarrhoeal Disease Research, Bangladesh (icddr,b), and downloaded the relevant information. We searched for unpublished grey documents in the repositories of the Expanded Program of Immunization (EPI), the Directorate and General of Health Services (DGHS), the WHO Bangladesh office, and other individuals related to polio eradication initiatives in Bangladesh. This literature review included grey documents on the polio program in Bangladesh since 1988 till 2018. Documents that addressed GPEI support in Bangladesh, effects for GPEI roll-out/impacts in Bangladesh, polio vaccine, polio-related fieldwork or lab work undertaken by GPEI partners, wild poliovirus and vaccine-derived polio studies, and polio surveillance were included, screened, translated if needed, extracted, and transferred into Qualtrics© (online survey software) for analyzing the data under different GPEI strategies, including setting up and mobilization, implementation, end-game strategies, impact on other health programs and health systems, facilitators, and barriers. The researchers reviewed and crosschecked the extracted data among themselves for comprehensiveness and any duplication. However, only AFP surveillance-related data were used for this manuscript.

We also conducted a quantitative survey with the members of Bangladesh’s ‘Polio Universe’, directly involved in polio eradication activities in Bangladesh for a minimum of 12 or more months between 1988 and 2019, from the EPI database, and additional respondents identified through snowball sampling. The questionnaire was designed by the STRIPE project team based on the Consolidated Framework for Implementation Research (CFIR) and the socio-ecological model [17] (Table 1). The Bangladesh team piloted the questionnaire and made a few adjustments to make it ready to use in Bangladesh context. A research team was sent to six administrative divisions of Bangladesh (i.e. Chittagong, Dhaka, Khulna, Rajshahi, Rangpur, and Sylhet Divisions) to collect data for paper-based surveys. The survey data were translated into English, cleaned, and analyzed using Stata 13. It was ensured that the full definition of each response (Table 1) was read and explained as it was written before the respondent answers.

**Table 1.** Overview of CFIR domains, indicating facilitators of and barriers for the Polio programme implementation in Bangladesh.

Domains	Characteristics of individuals within your organization	Organizational settings/Inner setting	Program/Intervention characteristics	Process of conducting the activities/implementation	External settings
Definitions	These include a low level of competence of coworkers within the respondent’s organization.	These include the environment and actions of the organization the respondent belongs to.	This includes medical products or technologies adopted by the organization and the actual strategy itself.	This includes strategies for how the program itself was conducted, including monitoring and evaluation activities.	External settings are any environment outside of the direct control of the project.
Determinants	● The person’s knowledge and beliefs about the activity ● Self-efficacy ● Individual stage of change ● Individual identification with organization	● Structural characteristics ● Networks and communications ● Culture: norms, values, basic assumptions of an organization led to challenges ● Implementation climate ● Readiness for implementation	● Intervention source ● Evidence strength and quality ● Relative advantage ● Adaptability to local context ● Trialability ● Complexity ● Design Quality and Packaging ● Cost	● Planning ● Engaging ● Executing ● Reflecting and evaluating	● Political environment ● Economic environment ● Social environment ● Technological environment ● Other environment

We conducted KIIs following the survey with a subset of survey participants, considering their expertise in polio eradication activities and survey nominations. Informed written consent was obtained, and interviews were recorded, transcribed, translated, and checked for any errors. We developed a priori codebook for deductive coding, following the CFIR and the socio-ecological model. A data display matrix was also developed to summarize the findings including relevant quotes from the participants and emerging codes were added to priori codes. Later, thematic analysis was performed to describe the evolution of AFP surveillance in Bangladesh, its facilitators, and barriers and additional activities leveraged from implementing the AFP surveillance programme. Ethical approval was obtained from the Institutional Review Board of the BRAC James P. Grant School of Public Health, BRAC University.

## Results

We identified total 92 literature and included 69 documents and excluded 23 following the inclusion and exclusion (Not related to Bangladesh, clinical, and lab-based paper) criteria. The grey documents we selected for data extraction include bulletins, reports, fact sheets, scientific papers, manuals, newspapers, and PowerPoint presentations. Table 2 describes the language, type, and source/publisher of the grey literature. This is to be informed that some of the grey literature have multiple sources or publisher.

**Table 2.** Categories of the identified grey literature.

Category	Frequency n = 92	Percent
**Language**		
Bangla	7	7.6
English	85	92.4
**Type of document**		
Bulletin	13	14.1
Scientific journal	8	8.7
Report	5	5.4
Fact sheet	6	6.5
Newspaper	2	2.1
Power point presentation	4	4.3
Manual	4	4.3
Protocol	1	1
Other	49	53.3
**Source/publisher of the literature**		
Government	61	66.3
GPEI core partners	40	43.5
Academic organization	8	8.7
Funding and implementing organization	31	33.7
Other	24	26

*Some of the literature has multiple source/publisher.

One hundred and twenty people were approached for survey and one hundred nine (90.8%) among them participated in survey, of whom 83 completed the paper-based survey, and 26 completed online (Table 3). Most respondents had more than ten years of experience with polio, and the maximum respondents worked in Government agencies (62.3%, *n* = 104) and WHO (17.4%, *n* = 29), respectively. The majority conducted vaccination (20.8%, *n* = 53), surveillance (17.3%, *n* = 44), and strengthening the delivery system (15.7%, *n* = 40), respectively. However, many of the respondents have conducted multiple roles in polio programme of Bangladesh and only data were collected who worked for AFP surveillance.

**Table 3.** Profile of the survey respondents.

Variables	Frequency n = 109	Percent
**Years involved with polio eradication activities**		
<5 years	16	14.7
6–15 years	29	26.6
16–25 years	43	39.4
25+	21	19.3
**Level in polio eradication activities**		
Global	18	10.1
National	46	25.8
District	66	37.1
Sub-district	48	27.0
**Organization affiliation**		
WHO	29	17.4
Rotary International	2	1.2
UNICEF	7	4.2
Center for Disease Control, US	3	1.8
NGO Implementing Partners	7	4.2
Government	104	62.3
Academic/research institution	4	2.4
Other	11	6.6
**Primary role in polio eradication activities**		
Resource mobilization	5	2
Partnership/alliance development	15	5.9
Strategy development and planning	29	11.4
Strengthening delivery systems	40	15.7
Vaccination	53	20.8
Surveillance	44	17.3
Community engagement	30	11.8
Monitoring & Evaluating	32	12.5
Others	7	2.7

*Many respondents served multiple roles in polio eradication activities.

Seventeen KIIs were conducted at the national level, and one at the sub-national level. Half of the KII participants were from the government, followed by other partners. Among the participants, 13 of them were involved in AFP surveillance.

The following themes emerged from the analysis of the grey literature review, survey, and KII findings.

### Evolution of AFP surveillance in Bangladesh

The AFP surveillance in Bangladesh was introduced in 1990 through the existing government structure. The Government assigned the Upazila Health and Family Planning Officer (UH&FPO) and the Civil Surgeon (CS) as Disease Surveillance Focal Persons (DSFP) at Upazila and district levels, respectively, and Medical Officer for Disease Control as Hospital Surveillance Officer (HSO) and Local Surveillance Officers (LSO) [19,20]. There were two surveillance systems including active surveillance, and passive surveillance system. Due to low reported cases, Government assigned volunteers and Non-government organization (NGO) workers in Upazilas and districts in 1996 for active case findings [20]. In urban areas, international and national organizations, including the United States Agency for International Development (USAID), BASICS, Immunization and Other Child Health (IOCH), Urban Primary Health Care Project (UPHCP), and Stop Transmission of Polio (STOP) program supported the primary surveillance system [21]. Finally, the country established a comprehensive AFP surveillance in 1997. However, the country is maintaining a standard AFP surveillance performance (Table 4) that is detection of >2 non-polio cases per 100,000 population <15 years of age since 2002 [22].

**Table 4.** AFP surveillance performance indicators Bangladesh, 2002–2016.

Indicators	2002	2003	2004	2005	2006	2007	2008	2009	2010	2011	2012	2013	2014	2015	2016
AFP rate	2.70	2.03	2.31	2.66	2.91	3.25	3.25	3.1	2.61	3.11	2.98	2.65	2.74	2.78	2.85
Non-polio AFP rate*	2.70	2.03	2.31	2.66	2.87	3.25	3.25	3.1	2.61	3.11	2.98	2.65	2.74	2.78	2.85
*%* adequate stool specimen collection*	89%	89%	90%	92%	93%	92%	92%	92%	94%	95%	96%	96%	98%	97%	99%
% Nonpolio enterovirus isolation	28.0	23.0	20.0	20.0	14.8	15.0	15.0	23.1	19.4	18.0	13.8	18.8	22.6	20.4	21
% timeliness of primary result reported	100	99	100	100	98	100	100	95	97	98	93	97	97	98	96

*Number of discarded AFP cases per 100,000 children <15 years of age.

*Percent with 2 specimens, 24 hours apart and <14 days of paralysis onset.

Source: EPI and VPD Surveillance Review and Post-Introduction Evaluation of Hib (Pentavalent) Vaccine (2012) and Polio Transition Plan Bangladesh.

WHO created the post of Surveillance Medical Officer (SMO) network in 1999 supported by GPEI. In 2002, District Immunization Medical Officer (DIMO) recruited by EPI merged with SMOs to form Surveillance Immunization Medical Officer (SIMO) [22]. Forty-four survey respondents and all KII respondents accredited the WHO’s contribution and their SMO network for strengthening the AFP and VDP surveillance.

The survey findings noted that the Virology Department of the Institute of Public Health (IPH) was the National Polio Laboratory (NPL) to diagnose polio cases, established in 1995 and is now an WHO-accredited laboratory in Bangladesh. In 2005, it included measles and rubella surveillance and was re-designated as the National Polio and Measles Laboratory (NPML).

Bangladesh achieved certified standards for ten AFP surveillance performance indicators in 2001. Due to effective AFP surveillance, Bangladesh detected 18 imported cases of wild poliovirus type 1 (WPV1) in 2006 from western Uttar Pradesh, India and contained the outbreak through extensive surveillance and supplementary immunization activities (SIAs) [22].

We do not have any indigenous cases since August 22, 2000. However, India was a threat to Polio importation. The newly detected cases from were restricted through surveillance, all surrounded people were vaccinated. KII_G66.

The survey and KII data found that environmental surveillance (ES) was established in September 2015 in Dhaka and Gazipur district and in 2018, extended to Cox’s Bazar district. No wild poliovirus cases in Bangladesh since 2006 and no isolations of wild poliovirus since the establishment of environmental surveillance.

**Figure 2.**
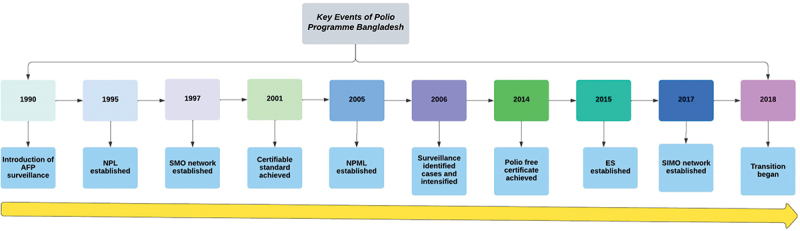
Key events of the polio program, Bangladesh.

For more than ten years, Bangladesh has achieved and maintaining a certificate standard of AFP surveillance performance indicators with a stool sample collection rate of 99%. Currently, 162 active surveillance and 787 passive surveillance sites are functional [12,23]. The key events of the polio program in Bangladesh are shown in Figure 2.

*NPL (National polio laboratory), SMO (Surveillance medical Officer), NPML (National Polio and Measles laboratory), ES (Environmental surveillance), SIMO (Surveillance immunization medical Officer).

### Role of the AFP surveillance in polio eradication of Bangladesh

**Figure 3.**
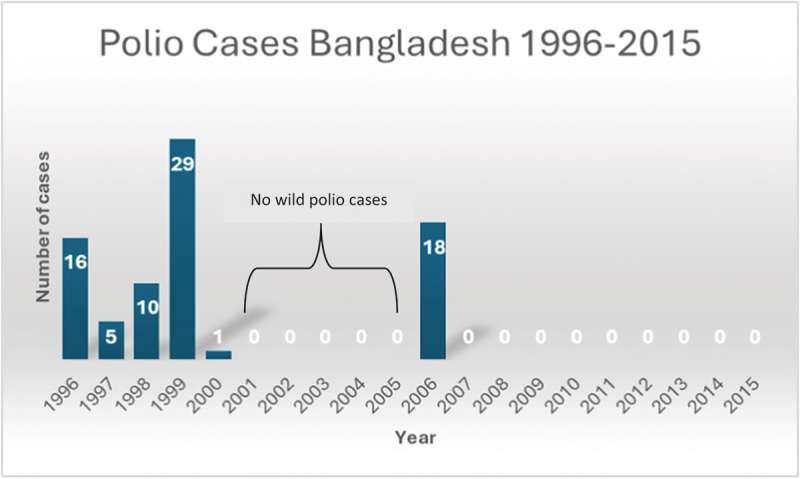
Polio cases in Bangladesh, 1996–2015.

When asked about the responsibilities of the survey respondents in polio eradication programme in Bangladesh, 44 respondents among 109 chose AFP surveillance as their primary responsibility reflecting the significant emphasis given to conducting AFP surveillance. The free text of the survey and the KII also highlighted that AFP surveillance led by the government under the Expanded Program on Immunization (EPI) and supported by the WHO successfully contributed to the decrease in poliomyelitis cases (Figure 3) and in attaining polio-free status for Bangladesh. One of the key informants also described the importance of AFP surveillance,

Since I started working, if I speak about the most critical two phases of polio eradication work, one would be the intervention that is the vaccination, and another is the searching for polio patients through surveillance. The surveillance still exists, and we still search for AFP cases, and the credit goes to EPI & SMOs said KII_G36.

### Key factors driving the success of AFP Surveillance

#### Multi-sectoral collaboration, support, and activities

**Figure 4.**
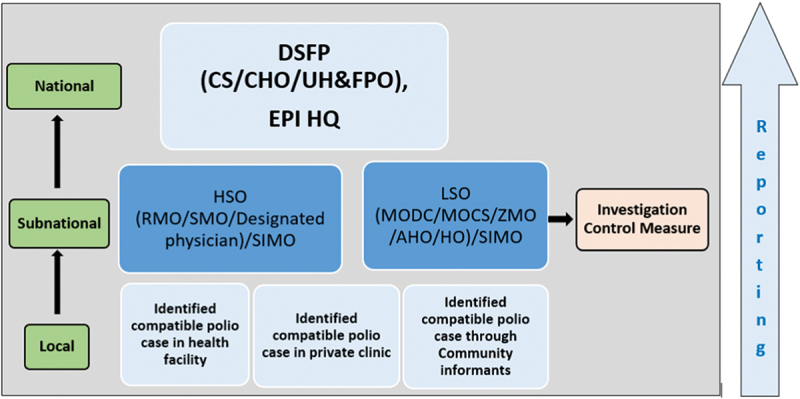
The structure and reporting system of the polio program in Bangladesh.

Nearly half (*n* = 18) of survey respondents said that process of conducting the activities (implementation, including the planning, execution strategies, reflection, and evaluation of activities, or adjustments made to the plan) was the main success factor for AFP surveillance (Table 5). This reflects the collaborative effective strategies of the government, EPI, the GPEI core partners, and other international and national NGOs contributed to the success of the AFP surveillance. Both free survey responses and KIIs (Figure 4) signify that the strong collaboration and social engagement among all relevant ministries, local Government, political leaders and parties, civil society, and local NGOs, professional EPI workforce, polio laboratory team, WHO SMO network, provided technical and financial support, facilitated the implementation, and kept Bangladesh polio-free. One KII responded said,

*DSFP (Disease Surveillance Focal Persons), CS (Civil Surgeon), CHO (Chief Health Officer), EPI HQ (Expanded Programme of Immunization Head Quarter), HSO (Hospital Medical Officer), LSO (Local Surveillance Officers) RMO Residential Medical Officer, SMO (Surveillance Medical Officer), SIMO (Surveillance Immunization Medical Officer), MODC Medical Officer for Disease Control, MOCS Medical Officer for Disease Surveillance, ZMO Zonal Medical Officer, AHO Airport Health Officer, HO (Health Officer).

International organization like GAVI, UNICEF, WHO…., altogether worked for polio, and they never delayed any EPI related activities. KII_G35. Another respondent stated,

Initially the task was started by the health ministry, but later to mobilize the community volunteers like Imams, Headmaster, other ministries like health, religion were involved. KII_NG55.

**Table 5.** Contributing factors and challenges of AFP surveillance implementation (survey).

Variables	Frequency (N)	Percentage (n %)
Contributors of Surveillance		
Process of conducting the activities/implementation	18	43.90
Program/Intervention characteristics	13	31.71
Organizational settings/Inner setting	7	17.07
Characteristics of individuals within your organization	3	7.32
External settings		
Social environment	22	53.66
Technological environment	11	26.83
Political environment	7	17.07
Economic environment	1	2.44
Challenges of Surveillance		
External settings (Social, technological, political, and economic environment)	16	38.10
Process of conducting the activities	14	33.33
Organizational settings	5	11.90
Polio eradication program characteristics	5	11.90
Characteristics of individuals within your organization	2	4.76

*The survey questionnaire has been subjected to multiple answers.

#### The SIMO network

The second highest, 31.71% (*n* = 13) survey respondents selected polio eradication program characteristics s. The WHO SMO network’s tremendous technical support, including identifying potential AFP cases, preserving, and transporting the sample, reporting to relevant bodies and follow-up, was also accredited by all the KII respondents. One of them described them as ‘the backbone of polio eradication in Bangladesh’ KII_G66. The grey literature review found similar findings [12,19,20].

Again, the literature [19] and the KIIs describe the role of SIMOs in strengthening the RI, developing EPI guidelines, assisting natural disasters and executing emerging and re-emerging infectious diseases surveillance. One KII respondent said,

Gradually, we (SMOs) entered the EPI. So, now in new vaccine introduction, RI monitoring, then rapid convenience, and data quality assessment, our SMOs are involved. Another respondent said,

AFP surveillance, and the surveillance for measles-rubella, NNT, Japanese Encephalitis JE, AEFI, and other VPDs are now implemented by the government with the assistance of SIMO KII_G58.

#### Social Environments

Most (53.7%, *n* = 22) of the survey respondents mentioned that the community setting, and context were major contributing external factors to successful AFP surveillance (Table 4). The nationwide community participation during National immunization days (NID)s and Supplementary national immunization days (SNID)s had high visibility in the polio eradication program. To increase the case notification and awareness, the Government trained and mobilized community volunteers and local NGO workers in Upazilas and districts named ‘key informants’. These informants were front-line primary health care workers, BRAC volunteers, Imams of local mosques, Union Council members, village doctors, local healers, EPI outreach sites caretakers, ORS and contraceptive depot holders, school teachers, scouts [21]. All KII respondents acknowledged their valuable contribution identifying AFP cases. One of the respondents said,

EPI did have limited staff to conduct the NID and child-to-child search. There were around six lakh volunteers, from community and people from different sectors… . they were very committed. KII_G55. Another respondent said,

Their contribution is excellent … If they did not give information, we would not have been able to strengthen surveillance. They are the real ones… . KII_G64.

#### High political commitment

Since 1971, political instabilities have taken place, including conflicts, strikes and shutdowns threatening the overall development of the country. Fortunately, the Government was highly committed to polio eradication efforts, including AFP surveillance from the beginning [24,25]. Around 17% of survey respondents informed political factors, which includes any political or high-level support either from local or national government, as a primary contributor. One of the study respondents stated,

The highest level of political commitment had a special focus on polio eradication. Due to political support, we did not face any difficulty in getting adequate support at political support from all government levels… . Every Government owned the program KII_G54.

### Challenges

In our survey, the majority 38.1% (*n* = 16) of respondents identified external factors as key challenges, which include political, economic, social, technological environment and others followed by the process of conducting the activities (33.3%, *n* = 14) and organizational settings (12%, *n* = 5) (Table 4). From the literature review and KII (Figure 4), the following external factors as challenges stood out:

#### Population density

Bangladesh is one of the most densely populated countries in the world, which is a significant challenge for planning AFP surveillance in the country, according to the grey literature [21]. The respondent rightly said,

And in our country, where the population is much big, it was difficult to reach all people. KII_NG60.

#### Hard to reach area

In the hard-to-reach areas include areas isolated by rivers, forests, large water bodies (‘Haors’), and hills. Many of them remain separated for months by floodwaters. The grey literature review noted that it was always difficult for health care providers to access and conduct regular reporting those remote areas with little or no health infrastructure support and active search for AFP cases [5,26]. Respondents to the KII expressed similar experiences:

Many places of Noakhali, Chittagong, Barishal, Haor areas have hard to reach areas where communicating is really hard. The surveillance team gave extra support there through. KII_NG56.

#### Geography

Bangladesh shares borders with India and Myanmar and has refugee camps near the Myanmar border. Bangladesh had no polio cases since 2000; however, 18 cases of Wild Polio Virus 1 (WPV1) were reported in 2006 imported from India [22]. India was polio-endemic until 2014, and chance of circulation was alarming. Myanmar had an outbreak of cVDPV on 7 November 2015 [27], posing a potential threat to importation [28]. Rohingya refugees labelled by the government as Forcibly Displaced Myanmar Nationals (FDMN) live in camps in Cox’s Bazar district in southeast Bangladesh [29] who pose an additional challenge to Bangladesh.

Similarly, one KII respondent mentioned,

Bangladesh is surrounded by India and Myanmar, so there was always a chance of importation. We do not have any indigenous cases since August 22, 2000. However, India has long been in endemic status till 2011. There was always a threat to the importation of polio. Therefore, once there is any case detection in the neighbouring country through surveillance, surrounded people were vaccinated. KII_G66.

#### The population at risk

The government conducted special microplanning for disadvantaged groups, including communities like snake-charmers, people living in boats and on islands (‘chars’) within rivers, teagarden workers, brothel inmates, minority groups, slum people, children of working mothers, and the floating population [23]. During Polio SIAs, vaccinators searched house to house and did queries about new AFP cases, especially in high-risk areas [5].

#### Transition

As we are close to eradicating polio globally, the GPEI has started a gradual transition with national partners globally. According to the transition plan started in 2019, AFP surveillance will be fully funded by the government, which will replace the SIMO network, and new epidemiologist/public health positions will be created by the government [22]. Also, one KII respondent said,

Now Bangladesh Government plans to absorb this network in some way. Ultimately the Government will take over the SIMO network according to the polio transition plan. KII_NG60.

However, in literature, free text of the survey, and KII response, uncertainty in implementing the plan and maintaining the current quality surveillance have emerged [22]. One KII respondent said,

Technical knowledge is not built in one day. It is not the time to stop the donor support in Bangladesh as the Government might not run these programs alone … KII_NG60. Another respondent described,

Without SMOs, AFP surveillance would not be the same KII_G54.

### AFP surveillance platform beyond Polio

Bangladesh also started the AFP surveillance platform beyond the polio program to other health initiatives, especially in other disease surveillance like surveillance for measles-rubella, NNT, Japanese Encephalitis JE, AEFI, and will continue stretching far through the transition plan. For emergencies, the SIMO network has been regularly utilized in different national emergencies and natural disasters and cyclones such as cyclones Sidr (2007), Aila (2009), and the 2004 tsunami and regular floods in the past two decades [12,22]. Also, in the COVID-19 crisis in Bangladesh, SIMOs were deployed to support all aspects of the COVID-19 response in their representative districts. They helped coordinate field activities, capacity building in Infection Prevention & Control, and contact tracing ensured prompt and safe transportation of laboratory samples and other supplies, conducted surveillance and resumed lifesaving vaccination throughout the country [11]. One of the KII respondents said,

Now there are lots of government program programs like Malaria program, the Rohingya program, nutrition program where the same health worker is responsible for giving support, who worked for polio. KII_G60.

Gradually, we (SMOs) entered the EPI. So, now speaking about new vaccine introduction, routine EPI monitoring, then rapid convenience assessment, data quality assessment our SMOs are involved in Measles elimination, Rubella CRS control, tetanus elimination status should be maintained till certification by us (SMOs).*”* KII_NG55

## Discussion

Though Bangladesh was the last country to eradicate smallpox in the world [30], it pioneered polio eradication in the Southeast Asian region by stopping polio transmission in 2000 [23]. This study highlights the valuable role of AFP surveillance in getting polio free status, the success of AFP surveillance depended on multi-sectoral collaboration and support, the SIMO network, community participation, and high-level political commitment. This study also focuses on challenges of AFP surveillance including reaching hard-to-reach areas and people, and transition of polio programme.

The study emphasizes on the central role of AFP surveillance played a crucial to gain the polio free status of Bangladesh through serving as an early warning system for the presence of the poliovirus. Maintaining sensitive AFP surveillance globally is vital to achieving and sustaining global polio free status [31]. In addition, low surveillance sensitivity is one of the main reasons for the reemergence of WPV in South-Eastern Africa in 2022 [32], which again implies the importance of this surveillance.

Robust multi-sectoral collaboration between ministries, the involvement of other stakeholders like NGOs and development partners and strong political commitments were among the contributors to the polio program in Bangladesh. Multi-sectoral intervention and coordination with collaborative planning and executing across different sectors at national, and local levels in Bangladesh can be seen in non-communicable disease sectors and nutrition as well [33,34]. During the recent battle against COVID-19, multi-sectoral collaboration among different ministries of Bangladesh and various national and international stakeholders reinforced the shield against the coronavirus [35]. Again the role of strong political commitment on strengthening AFP surveillance is also evident in country like India and Nigeria [36,37]. In addition, the support from the partners and donors helped reduce the incidence of polio globally by 99% since the launch of GPEI [38].

The study identifies technical tasks including AFP case detection, sample collection, and testing, coverage evaluation surveys to measure OPV 3 coverage, provided an evidence-based approach to the success of the surveillance system and other vaccine-preventable diseases. Among the GPEI core partners, WHO provides the bulk of technical assistance mentioned above to the Government in the setting up and functioning of the polio surveillance at national and sub-national levels and also supports the quality assurance and standardization of surveillance laboratories [26, 28]. WHO provided technical support to the Ministry of Health, Bangladesh through building a well-established polio surveillance system through the SIMO network, which helped to combat polio and other emerging and reemerging diseases including measles-rubella, Neonatal Tetanus NNT, Japanese Encephalitis JE, malaria, and Kala-azar [29]. Like Bangladesh, Nepal and Somalia is also using the AFP surveillance for VDPs through WHO-supported national program, in [39,40]. In addition, SMOs from the National Polio Surveillance Project (NPSP) of India played a crucial role in surveillance activities both at the national and state level [41].

The study shows that in Bangladesh, key informants or community volunteers were involved in identifying and notifying AFP cases to the authority as part of community surveillance. Engaging communities against Polio was also adopted in many African countries, including Nigeria, Somalia, Kenya, Ethiopia, and Zimbabwe [6,40–43]. In Afghanistan, ‘mullahs’ (religious leaders), traditional healers, healthcare providers, teachers, parents, and others are trained to detect AFP cases in their community [44].

This study revealed that the low performance of AFP surveillance initially, in hard-to-reach areas, and among the ‘floating’ and dense population, transition and importation threat were barriers to implementing the AFP surveillance. These challenges match with other countries as well. The poliovirus remains a major challenge for remote and inaccessible places worldwide. Some areas are sparsely populated, some are densely packed, and the river beds of Lake Chad and some are in conflict-affected countries [45]. Additionally, WHO says that funding decrease will increase the risk of disrupting the main EPI functions, especially the disease surveillance due to polio transition planning [46]. Similarly, in Bangladesh, the polio transition poses risks and challenges in implementing the plan of substituting the staff, resulting in compromised surveillance. In addition, as the development partners supported most of the tasks and funding, it will be crucial for Bangladesh to sustain the standard surveillance system [22]. However,

three countries that are Bangladesh, India, and Indonesia among other priority countries in the Southeast Asian Region, are advanced in implementing the national transition plans along with funding support from the governments [47].

The study also identifies that in Bangladesh, an extensive set-up of the national disease surveillance system and a national laboratory are established with the support of GPEI identifying the importance of moving forward for attaining different global and national goals of disease elimination and control, particularly VPDs. nearly. Multiple disease surveillance interventions are integrated with AFP surveillance, which has become a standard practice in many countries for measles and rubella surveillance [8,10]. According to a survey conducted in 2000, 26 of the 32 countries used AFP surveillance resources for other infectious diseases like measles, neonatal tetanus, cholera, meningitis, and yellow fever [48]. Similarly, by 2003, 131 of 198 countries globally were using the AFP surveillance system for surveillance of other VPDs [8].

## Conclusions

This manuscript provides a systematic exploration of the AFP surveillance system in Bangladesh since its inception. The study identifies key facilitators including collaboration between government entities, national and international organizations, and community volunteers. This also identifies challenges including population density, reaching remote areas, geopolitical considerations, and the ongoing transition of AFP surveillance to the government. The manuscript also emphasizes that as the world is so close to eradicating polio, the assets, knowledge, facilitators, barriers, and additional functions of the AFP surveillance platform\, could be used and transferred to other health programmes in other countries as well. Therefore, it is high time for the AFP surveillance team to provide guidance and support to the Government to create a surveillance plan for emerging infectious diseases, train and sensitize health workers, and support the laboratories. The government should recognize the value of polio transition plan and secure the ownership over the programme and funding and strengthen their technical capacity for sustaining the polio free status and smooth transitioning of polio assets to other health priorities.

## Data Availability

Data and publications from this project will be Open Access and available via an online repository.
